# Changes in Inflammatory Markers in Patients with Chronic Thromboembolic Pulmonary Hypertension Treated with Balloon Pulmonary Angioplasty

**DOI:** 10.3390/cells11091491

**Published:** 2022-04-29

**Authors:** Wojciech Magoń, Jakub Stępniewski, Marcin Waligóra, Kamil Jonas, Roman Przybylski, Piotr Podolec, Grzegorz Kopeć

**Affiliations:** 1 Department of Cardiac and Vascular Diseases, John Paul II Hospital in Krakow, 31-202 Krakow, Poland; magon.wojtek@gmail.com (W.M.); jakub.stepniewski@googlemail.com (J.S.); marcin.waligoora@gmail.com (M.W.); kamil.jns@gmail.com (K.J.); ppodolec@interia.pl (P.P.); 2 Pulmonary Circulation Centre, Department of Cardiac and Vascular Diseases, Faculty of Medicine, Jagiellonian University Medical College, 31-008 Krakow, Poland; 3 Center for Innovative Medical Education, Department of Medical Education, Faculty of Medicine, Jagiellonian University Medical College, 30-688 Krakow, Poland; 4 Institute of Heart Diseases, Department of Cardiac Surgery and Heart Transplantation, Wroclaw Medical University, 50-367 Wroclaw, Poland; przybylski240@gmail.com; 5 Department of Cardiac and Vascular Diseases, Faculty of Medicine, Jagiellonian University Medical College, 31-202 Krakow, Poland

**Keywords:** biomarker, chronic thromboembolic pulmonary hypertension, endothelin 1, interleukin 6, interleukin 8, endothelial function

## Abstract

Background: Inflammatory response and endothelial dysfunction contribute to the progression of chronic thromboembolic pulmonary hypertension (CTEPH). We aimed to assess changes in biomarkers involved in those processes in inoperable CTEPH patients treated with balloon pulmonary angioplasty (BPA). Methods: We enrolled 20 patients with inoperable CTEPH qualified for BPA and a control group. Interleukin 6, 8, 10 (IL-6, IL-8, IL-10), monocyte chemoattractant protein-1 (MCP-1), and C-reactive protein (hsCRP) constituted the markers of systemic inflammation. Endothelin 1 (ET-1) served as a marker of endothelial dysfunction. Selected markers were assessed before the BPA treatment, 24 h after the first BPA, and six months after completion of the BPA treatment. Results: At baseline, the CTEPH patients had increased serum concentrations of IL-6, IL-8 and ET-1. Twenty-four hours after a BPA session, we observed an increase in concentrations of IL-6 (∆ = 3.67 (1.41; 7.16); *p* < 0.001), of IL-10 (∆ = 0.25 (0; 0.47); *p* = 0.003), of MCP-1 (∆ = 111 (60.1; 202.8); *p* = 0.002), and of hsCRP (∆ = 4.81 (3.46; 8.47); *p* < 0.001). Six months after completion of the BPA treatment, there was a decrease in concentrations of IL-6 (∆ = −1.61 (−3.11; −0.20); *p* = 0.03), of IL8 (∆ = −3.24 (−7.72; 0.82); *p* = 0.01), and of ET-1 (∆ = −0.47 (−0.96; 0.05); *p* = 0.005). Conclusions: Patients with inoperable CTEPH exhibit increased systemic inflammation and endothelial dysfunction, which improves after completion of the BPA treatment. A single BPA session evokes an acute inflammatory response.

## 1. Introduction

Chronic thromboembolic pulmonary hypertension (CTEPH) is a rare and progressive disease caused by the obstruction of pulmonary arteries by unresolved organized thrombi with accompanying precapillary arteriopathy [1,2,3,4].

The exact pathogenesis of CTEPH has not been fully explained, although it has been shown that development of CTEPH involves various mechanisms leading to incomplete resolution of thrombi and sustained pulmonary vascular remodeling [5]. Some data suggest that excessive inflammatory response [6] and endothelial dysfunction [7] contribute to progression of the disease. Circulating levels of several inflammatory cytokines are increased in CTEPH patients [8] and in part reflect the local inflammation found in the organized thrombi removed during pulmonary endarterectomy (PEA) [6]. The increased levels of cytokines together with overproduction of endothelin 1 (ET-1), a potent vasoconstrictor and a marker of endothelial dysfunction, correlate with the clinical severity of CTEPH [9].

Currently pulmonary endarterectomy (PEA) remains the treatment of choice for most patients with CTEPH [1], and balloon pulmonary angioplasty (BPA), along with PH-specific drugs, are appropriate treatment modalities for inoperable patients [10,11,12,13,14,15]. Unlike PEA, which is usually curative as a single procedure, BPA needs to be divided into a series of sessions to prevent lung injury. Previous studies have described that ET-1 levels before PEA, and inflammatory status after PEA [16], affect the outcome of this treatment [17,18]. To our knowledge, there are no studies concerning changes in the inflammatory profile and endothelial function after BPA treatment.

In the current study, our aim was to evaluate changes in plasma biomarkers of inflammation and of endothelial function in CTEPH patients treated with BPA after a single BPA session, and in the long-term follow-up after completion of interventional treatment.

## 2. Materials and Methods

### 2.1. Study Population

The study group consisted of adult, inoperable CTEPH patients who had undergone complete BPA treatment between 2015 and 2017 in a single pulmonary hypertension (PH) reference center. We started to enroll consecutive patients in the study after performing 10 BPA sessions in our center to exclude the effect of the learning curve on the results. The control group consisted of age- and sex-matched healthy individuals in which echocardiography revealed no significant abnormalities and a low probability of pulmonary hypertension.

### 2.2. Evaluation of Patients

Diagnosis of CTEPH was established according to the current guidelines [3], and operability and eligibility for BPA were assessed by the local CTEPH team, consisting of a cardiac surgeon, an interventional cardiologist experienced in BPA, and a PH specialist. Inoperability was determined based on correlation between hemodynamic parameters, pulmonary angiography, and other available imaging. Patients with thromboembolic lesions in segmental or more proximal pulmonary arteries were considered operable. Inoperable patients had more distal lesions or comorbidities significantly increasing the risk of surgery. In the CTEPH patients, we evaluated the World Health Organization functional class (WHO-FC), serum levels of the N-terminal prohormone of brain natriuretic peptide (NT-proBNP), 6-minute walking distance (6MWD), and the following hemodynamic parameters: mean pulmonary arterial pressure (mPAP), right atrial pressure (RAP), cardiac index (CI), and pulmonary vascular resistance (PVR). The parameters were acquired before the first BPA session (baseline), before each of the subsequent BPA sessions (before BPA), and six months after the final BPA session (6-month follow-up). Subjects in the control group were reviewed for the presence of any comorbidities that might affect pulmonary circulation, and transthoracic echocardiography was performed. The institutional ethics committee (Komisja Bioetyczna przy Okręgowej Izbie Lekarskiej w Krakowie) approved the study protocol, and written informed consent was obtained from each subject before starting the study. All clinical investigations were conducted according to the principles expressed in the Declaration of Helsinki.

### 2.3. Blood Samples

Venous blood samples of patients with CTEPH were collected, after an overnight fast, for the assessment of biomarkers concentrations at baseline, before a single BPA session, and 24 h after a single BPA session, when no periprocedural complications were noted. In cases where a significant periprocedural complication (PA dissection, clinical signs of lung injury) was recognized, blood was collected before and after the next consecutive BPA session without periprocedural complications. At follow-up, blood samples were collected, six months after the final BPA session.Serum concentrations of endothelin 1 (ET-1) (assay range: 0.3–250 pg/mL; coefficient of variation [CV]: 3.4%), of interleukin 6 (IL-6) (assay range: 0.2–10 pg/mL; coefficient of variation [CV]: 4.7%), of interleukin 8 (IL-8) (assay range: 1.0–64 pg/mL; coefficient of variation [CV]: 5.5%), of interleukin 10 (IL-10) (assay range: 0.8–50 pg/mL; coefficient of variation [CV]: 9.4%), and of monocyte chemoattractant protein 1 (MCP-1) (assay range: 31.2–2000 pg/mL; coefficient of variation [CV]: 4.2%) were analyzed using Quantikine ELISA kits (R&D Systems, MN, USA). Serum concentration of C-reactive protein (hsCRP) was determined in the routine diagnostic laboratory in our center (Roche, Basel, Switzerland) (assay range: 0.15–20 mg/L; coefficient of variation [CV]: 1.6%).

### 2.4. Balloon Pulmonary Angioplasty

Every BPA session was preceded by right heart catheterization [19] to assess the hemodynamic effect of the previous BPA session. It consisted of a series of balloon inflations (BPAs) of stenosed pulmonary artery segments, as described in detail in our recent report [11].

### 2.5. Statistics

The categorical variables are presented as counts and percentages, and the continuous variables as medians and interquartile ranges. To assess differences in serum concentrations of selected biomarkers between CTEPH group and controls, we used the Mann–Whitney U test. To assess differences in serum concentrations of selected biomarkers at each time point, and to assess the treatment effects of BPA for continuous clinical parameters, we used the Wilcoxon matched-pairs signed-rank test. To assess differences in categorical clinical parameters after BPA treatment, we used a χ^2^ test. To analyze the associations between each biomarker and the hemodynamic and clinical parameters, we used univariable linear regression models. The significance level was set at *p* < 0.05. The statistical analysis was performed with Stata/SE 12.1 StatCorp LP and RStudio version 0.99.467.

## 3. Results

### 3.1. Study Groups

We enrolled 20 patients with CTEPH, with median age of 67 (61–74) years, including six (30%) males. The control group consisted of 10 patients (three (30%) males) aged 58 (50–66) years, free of major cardiovascular or respiratory diseases. In the CTEPH group, 11 (55%) patients had been treated with stable doses of PH-specific medications before starting the first BPA session, including subcutaneous treprostinil (n = 5; 25%), riociguat (n = 4; 20%), sildenafil (n = 1; 5%), or a combination of subcutaneous treprostinil and riociguat (n = 1; 5%). Nine patients had not been pretreated with targeted therapies before BPA, as these therapies were not available at that time. One (5%) patient had persistent CTEPH after PEA. The characteristics of the enrolled patients with CTEPH are presented in Table 1.

### 3.2. Hemodynamic and Clinical Effects of BPA

The median time between the analyzed consecutive BPA sessions was 33 (21–47) days. The single sessions targeted a median of 5 (4–6) stenosed pulmonary arterial segments, and resulted in a decrease of PVR (1.2 ((−)0.9–2.8) WU, *p* = 0.04) but without a significant change of mPAP (−1 ((−)5–1) mmHg, *p* = 0.10), RAP (0.5 ((−)1–1.5) mmHg, *p* = 0.26) or CI (0.26 ((−)0.10–0.43) L/min/m^2^, *p* = 0.11).

The median number of BPA sessions per patient was six (3–8), and the total number of treated segments was 30 (20–40). The whole treatment resulted in an improvement of functional capacity, exercise tolerance, hemodynamics, and NT-proBNP levels (Table 1).

### 3.3. Baseline Levels of Selected Biomarkers in CTEPH Patients and Controls

The markers of inflammation showed a heterogeneous pattern (Table 2). As compared to controls, CTEPH patients had higher levels of IL-6 and IL-8, and similar levels of hsCRP, MCP-1 and IL-10. We also observed elevated levels of ET-1 in the CTEPH group.

In univariable linear regression models, the baseline serum concentration of ET-1 (but not other markers) was positively associated with baseline values of log NT-proBNP (β = 0.96 ± 0.23; *p* = 0.001, R^2^ = 0.50), mPAP (β = 5.0 ± 1.8; *p* = 0.01, R^2^ = 0.31) and PVR (β = 2.3 ± 0.8; *p* = 0.01, R^2^ = 0.32).

### 3.4. Changes in Biomarker Levels after a Single BPA Session

We analyzed serum concentrations of selected biomarkers before and 24 h after the first BPA session, in 16 (80%) patients. For the remaining four (20%) patients we assessed biomarkers during the second (three (15%) patients) and the third (one (5%) patient) BPA session due to procedure complications: two reperfusion lung injuries not requiring invasive ventilation, and two cases of hemoptysis requiring balloon occlusion of the segmental pulmonary artery.

**Figure 1:**
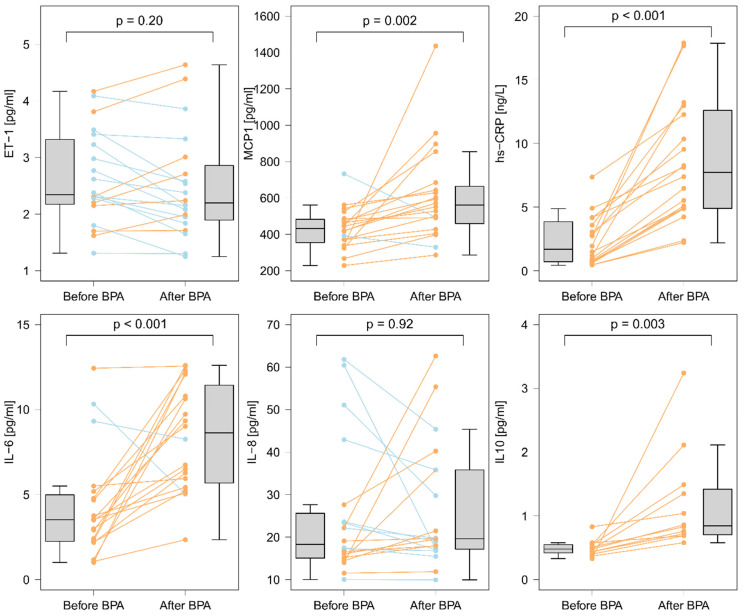
Serum concentrations of biomarkers before and 24 h after a single BPA session. There were increases in concentrations of IL-6 (∆ = 3.67 (1.41; 7.16); *p* < 0.001), of IL10 (∆ = 0.25 (0; 0.47); *p* = 0.003), of MCP-1 (∆ = 111 (60.1; 202.8); *p* = 0.002), and of hsCRP (∆ = 4.81 (3.46; 8.47); *p* < 0.001), and no change in IL-8 (∆ = 0.12 (−5.44; 5.23); *p* = 0.92), or ET-1 (∆ = −0.2 (−0.5; 0.23); *p* = 0.20). The orange color indicates an increase, and the blue color indicates a decrease. The difference between the two time points was determined using the Wilcoxon matched-pairs signed-rank test.

Twenty-four hours after BPA we observed an increase in the serum concentrations of IL-6, IL-10, MCP-1, and hsCRP, with no change in the concentrations of IL-8, or ET-1 (Figure 1).

### 3.5. Changes in Selected Biomarkers Levels after Completion of BPA Treatment

**Figure 2:**
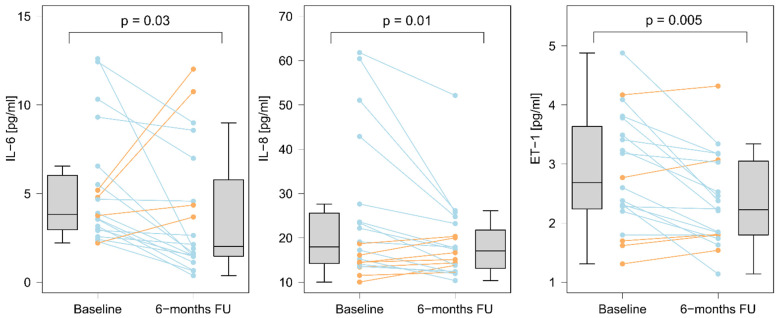
Serum concentrations of biomarkers before and after completion of BPA treatment. There was a decrease in concentrations of IL-6 (∆ = −1.61 (−3.11; −0.20); *p* = 0.03), of IL8 (∆ = −3.24 (−7.72; 0.82); *p* = 0.01), and of ET-1 (∆ = −0.47 (−0.96; 0.05); *p* = 0.005). The orange color indicates an increase, and the blue color indicates a decrease. The difference between the two time points was determined using the Wilcoxon matched-pairs signed-rank test.

**Figure 3:**
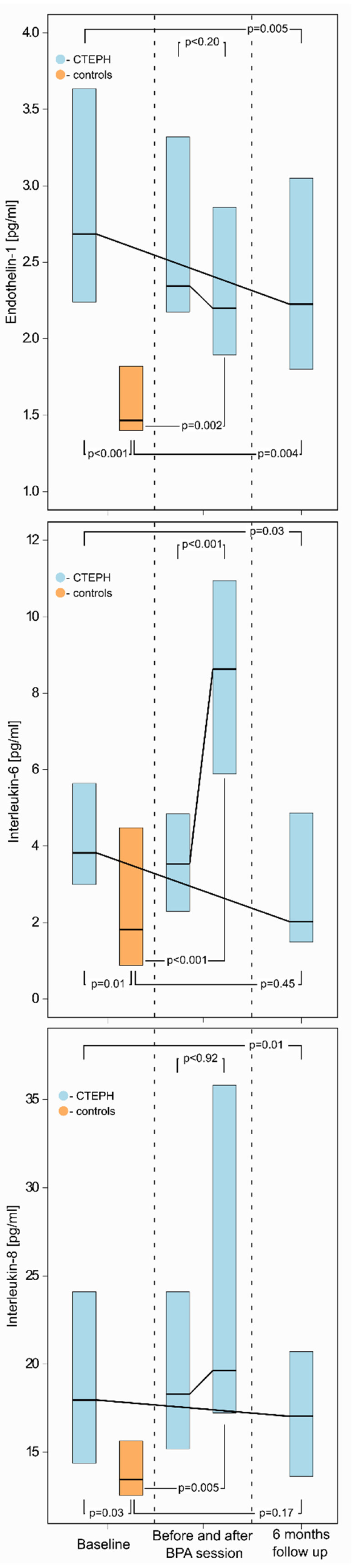
Serum concentrations of endothelin 1, interleukin 6, and interleukin 8 at different time points (baseline, after single BPA session, after completion of BPA treatment) during BPA treatment as compared to control group. IL-6 and IL-8 levels normalized after completion of BPA treatment. The difference between the two time points in the CTEPH group was determined using the Wilcoxon matched-pairs signed-rank test. The difference between the CTEPH group and the controls was determined using the Mann–Whitney U test.

We evaluated all patients, six (5–7) months after the last BPA session. Among the biomarkers that were increased at baseline evaluation (as compared to controls), we observed a decrease in the serum concentrations of ET-1, IL-6, and IL-8 after completion of BPA treatment (Figure 2 and Table S1). The final concentrations of IL-6 and IL-8 did not differ from the controls: 2.62 (1.49–5.78) vs. 1.81 (0.89–4.48), *p* = 0.45 and 17.04 (13.12–21.79) vs. 13.45 (12.46–16.01), *p* = 0.17, respectively. On the contrary, ET-1 concentrations after completion of BPA treatment remained elevated as compared to controls: 2.22 (1.80–3.05) vs. 1.47 (1.40–1.82), *p* = 0.004 (Figure 3).

### 3.6. Associations between Levels of Biomarkers and BPA Treatment Outcomes

#### 3.6.1. The Effect of a Single BPA Session

Periprocedural increase in inflammatory biomarkers after a single BPA session was positively associated with the hemodynamic improvement on follow-up RHC. Increases in IL-6, IL-10, and MCP-1 correlated with decreases in mPAP (β = 0.5 ± 0.2; *p* = 0.04, R^2^ = 0.25 for IL-6; β = 3.9 ± 1.2; *p* = 0.005, R^2^ = 0.36 for IL-10; β = 0.011 ± 0.003; *p* = 0.004, R^2^ = 0.37 for MCP-1) and PVR (β = 0.28 ± 0.10; *p* = 0.01, R^2^ = 0.29 for IL-6; β = 1.62 ± 0.59; *p* = 0.01, R^2^ = 0.29 for IL-10; β = 0.010 ± 0.001; *p* = 0.004, R^2^ = 0.42 for MCP-1). Moreover, an increase in IL-6 correlated with an increase of CI (β = 0.06 ± 0.02; *p* = 0.01, R^2^ = 0.33).

#### 3.6.2. The Effect of Completed BPA Treatment

A decrease in ET-1 positively correlated with the overall decrease in PVR (β = 3.2 ± 1.1; *p* = 0.01, R^2^ = 0.33), RAP (β = 3.4 ± 1.5; *p* = 0.04, R^2^ = 0.22), and log NT-proBNP (β = 1.1 ± 0.5; *p* = 0.04, R^2^ = 0.23). In addition, a decrease in IL-6 positively correlated with decreases in mPAP (β = 1.1 ± 0.4; *p* = 0.02, R^2^ = 0.27), and in PVR (β = 0.4 ± 0.2; *p* = 0.03, R^2^ = 0.26), and an increase in 6-minute walking distance (β = 8.7 ± 2.9; *p* = 0.008, R^2^ = 0.34).

## 4. Discussion

The present study analyzed, for the first time, temporal changes in inflammatory cytokines and ET-1 concentrations during BPA treatment in CTEPH patients. We showed that IL-6 and IL-8 were elevated in CTEPH patients as compared to controls, but they normalized after completion of treatment. The decrease of IL-6 was proportional to an improvement in hemodynamics and endothelial function. We also found an unspecific increase of several inflammatory markers immediately after a single BPA session.

Our study is of clinical significance, as the inflammatory status and endothelial dysfunction induce a prothrombotic state. Accordingly, the acute inflammatory response after a BPA session should be considered when deciding about the perioperative anticoagulation protocol, which currently is not established, and changes from center to center. Moreover, some reports suggest that treatment may be discontinued to reduce the risk of hemoptysis [20]. In our group, increase in cytokine levels did not have a negative effect on the hemodynamic results of BPA, which may be explained by the fact that all patients had uninterrupted oral anticoagulation and, if treated with vitamin K antagonists, the international normalized ratio (INR) was required to be between 2 and 3 on the day of the procedure. 

The acute inflammatory response was represented in our study by a rise of several interleukins but also CRP, which is a commonly used marker of inflammation in clinical practice. Its increase in combination with auscultatory signs of segmental lung reperfusion may be falsely interpreted as pneumonia, leading to unnecessary antimicrobial treatment. In our practice, we usually observe the patient, and do not use antibiotics if the CRP level decreases and the symptoms subside in a couple of days. Of clinical significance, also, is the long term anti-inflammatory effect of BPA, as inflammation, similarly to PAH [21], contributes to CTEPH pathogenesis and correlates with morbidity in this group of patients.

Previous studies have found elevated levels of several inflammatory biomarkers in patients with CTEPH, but the types of cytokines which were raised, or which had clinical significance, changed from one study to another. The cytokines in which plasma levels were shown to be elevated in CTEPH included IL-1β, IL-2, IL-4, IL-6, IL-8, IL-10, TNF-α, CRP, CXCL9, and MCP-1 [8,22]. In one study, both raised CRP and raised MCP-1 correlated with worse hemodynamic parameters [23]. In another study[24], raised levels of IL-8 and CXCL9 were associated with decreased survival in CTEPH patients. 

The dynamics of cytokines in treated patients had been evaluated in operable patients in the context of PEA. It was observed that, in this group, increased plasma levels of IL-6 and IL-8 predicted persistent PH after PEA [25], and that higher levels of CRP predicted poor early outcomes after PEA [26]. To the best of our knowledge, the changes in cytokine levels in inoperable patients treated with BPA have not yet been analyzed.

In our population of inoperable CTEPH patients, as compared to populations of previous studies, enrolling only operable or both operable and inoperable patients, we found similarly elevated levels of IL-6 and IL-8 but, unlike the other studies, we did not observe increased levels of hsCRP, IL-10, or MCP-1. This could be at least partly explained by the fact that patients who have an inoperable disease have different clinical characteristics from operable patients [2,27]. They are older, predominantly female, and have more comorbidities, including chronic kidney disease, which may impact the levels of various cytokines.

We have found a significant increase in most of the studied inflammatory markers (IL-6, IL-10, hsCRP, MCP-1) early after a single BPA session, which we interpret as an occurrence of acute inflammatory response. A similar observation was previously made in operable CTEPH patients undergoing PEA [28], where transient increases in IL-6 and IL-10 were also observed immediately after PEA with a rapid decrease to preprocedural levels after several hours. Of clinical importance is the fact that the acute increase in the levels of inflammatory markers observed in our study after BPA, correlated with the hemodynamic effectiveness of this procedure, as measured by a decrease in PVR. As BPA includes some damage to the arterial wall [29,30] and leads to the reperfusion of lung tissue, we speculate that the correlation between inflammatory response and decrease in PVR can be explained by the overall extent of the procedure. 

The normalization of the initially elevated levels of inflammatory markers after the completion of the whole BPA treatment indicates a potential therapeutic anti-inflammatory effect of BPA. This observation corresponds with other curative effects of BPA, including improvement in hemodynamics [12,31], right ventricular function [32], and physical capacity, which have anti-inflammatory potential, per se. Additionally, in-vitro studies on PEA tissue specimens [33] have shown that endothelial cells present in patients with CTEPH expressed basal pro-inflammatory status with increased production of inflammatory cytokines. We can speculate that improvement of hemodynamics decreases the damage to the PA wall caused by high blood pressure, resulting in a positive change of endothelial phenotype. This is further supported by the decrease of ET-1 levels observed in our study.

The decrease in the levels of ET-1 six months after treatment completion is a new finding, to our knowledge not previously described. Previous studies have shown higher levels of ET-1 and its possible impact on treatment outcomes after PEA [9,18]. ET-1 is an important biomarker associated with the pathogenesis of pulmonary arterial hypertension [34], and it is postulated that it contributes to pathological remodeling of pulmonary microcirculation [35] in the course of CTEPH. Although the levels of ET-1 decreased after completion of BPA treatment, it was still higher than in the controls. This may indicate the need for additional endothelium-targeted treatment in some patients.

### Strengths and Limitations

Most of our findings are novel, as we analyzed changes in various biomarkers after treatment with BPA for the first time. Additionally, we have discovered some new associations between levels of biomarkers and the clinical effectiveness of BPA. As summarized above, our study also has important clinical implications.

Our study also has some limitations. Firstly, our treatment group was heterogenous in terms of baseline treatment with PH-specific drugs, as approximately half of our patients were on treatment before BPA. However, we performed additional supplementary analyses in subgroups of patients, which did not reveal any significant discrepancies in baseline biomarker levels between patients who used PH-specific medications and those who did not (Table S2). Secondly, we arbitrarily used a period of six months of follow-up after the last BPA to assess its effectiveness, although this was because that particular period has been used in most of the previous studies regarding the effects of BPA treatment [36]. Thirdly, the size of our study group is small, but we made efforts to control the confounding factors by careful selection of analyzed procedures, as only sessions without complications were accepted for analysis.

## 5. Conclusions

In inoperable CTEPH patients, a single BPA session leads to an acute and unspecific inflammatory response. Long-term BPA leads to a significant reduction in the levels of circulating cytokines and improvement in endothelial function. The decrease of IL-6 is proportional to an overall hemodynamic improvement after a series of BPA sessions.

## Figures and Tables

**Table 1:** Clinical and hemodynamic characteristics of patients with chronic thromboembolic pulmonary hypertension (n = 20).

Variable	Before BPA Treatment	Six Months after Completion of BPA Treatment	*p*
Age, years	67 (65–75)	-	-
Male sex, n, %	6 (30%)	-	-
Time from onset of symptoms to CTEPH diagnosis, months	10 (5–22)	-	-
WHO functional lass: I/II/III/IV, n, %	0/0/20/0 0%/0%/100%/0%	2/12/6/0 10%/60%/30%/0%	<0.001
6-minute walking distance *, m	330 (260–380)	393 (340–450)	0.01
NT-proBNP, pg/mL	1726 (521–2678)	236 (144–722)	0.002
mPAP *, mmHg	39 (37–50)	29 (25–31)	<0.001
RAP, mmHg	4 (3–7)	4 (3–6)	0.26
CI *, L/min/m^2^	2.31 (1.95–2.62)	2.49 (2.32–3.00)	0.12
PVR *, WU	8.9 (6.3–11.1)	3.9 (3.5–5.7)	<0.001

Continuous variables are presented as medians and interquartile ranges. *—variable with normal distribution. Abbreviations: CI, cardiac index; CTEPH, chronic thromboembolic pulmonary hypertension; mPAP, mean pulmonary artery pressure; NT-proBNP, N-terminal prohormone of brain natriuretic peptide; PVR, pulmonary vascular resistance; RAP, right atrial pressure, WHO, World Health Organization.

**Table 2:** Comparison of serum concentrations of selected biomarkers between patients with CTEPH and control group.

	CTEPH (n = 20)	Controls (n = 10)	*p*
hsCRP, mg/L	2.16 (0.75–4.48)	2.15 (0.88–2.77)	0.79
IL-6, pg/mL	3.82 (2.96–6.03)	1.81 (0.88–4.48)	0.01
IL-8, pg/mL	17.96 (14.23–25.62)	13.45 (12.46–16.02)	0.03
IL-10, pg/mL	0.49 (0.39–0.58)	0.51 (0.48–0.69)	0.98
MCP-1, pg/ml	419 (338–506)	416 (273–505)	0.84
ET-1, pg/mL	2.68 (2.24–3.64)	1.47 (1.4–1.82)	<0.001

CTEPH, chronic thromboembolic pulmonary hypertension; ET-1, endothelin 1; hsCRP, high sensitivity C-reactive protein; IL-6, interleukin 6; IL-8, interleukin 8; IL-10, interleukin 10; MCP-1, monocyte chemoattractant protein 1.

## Data Availability

Data will be available upon request.

## Supplementary Materials

The following supporting information can be downloaded at: https://www.mdpi.com/article/10.3390/cells11091491/s1, Table S1. Serum concentrations of biomarkers at four specified time-points in the CTEPH patients. Table S2. Comparison of serum concentration of selected biomarkers between treatment-naive patients with CTEPH, patients with CTEPH on treatment, and control group.
