# Supplementary material for: Changes in Inflammatory Markers in Patients with Chronic Thromboembolic Pulmonary Hypertension Treated with Balloon Pulmonary Angioplasty
Source: Cells. 2022 Apr 29;11(9):1491. doi: 10.3390/cells11091491 (PMC9102042; doi:10.3390/cells11091491)
Supplement: Supplementary file 1 [file cells-11-01491-s001.zip › cells-1584758-supplementary.pdf]

Supplementary Tables:

Table S1. Serum concentrations of biomarkers at four specified time-points in the CTEPH patients.

	Before single BPA	24 hours after BPA	Baseline	6 months after treatment	p[a]	p[b]
ET-1	2.35 [2.18-3.32]	2.2 [1.9-2.86]	2.68 [2.24-3.64]	2.22 [1.8-3.05]	0.20	0.005
MCP-1	432.6 [354.9-482.6]	561.5 [459.5-663.9]	418.7 [338.2-506.1]	429.9 [372.9-508.7]	0.001	0.99
IL-6	3.53 [2.26-4.99]	8.63 [5.69-11.43]	3.82 [2.96-6.03]	2.02 [1.47-5.78]	<0.001	0.03
IL-8	18.29 [15.07-25.62]	19.63 [17.18-35.82]	17.96 [14.23-25.62]	17.04 [13.12-21.79]	0.91	0.01
IL-10	0.48 [0.39-0.58]	0.79 [0.63-1.45]	0.49 [0.39-0.58]	0.58 [0.32-0.77]	0.003	0.50
hsCRP	1.69 [0.71-3.85]	7.73 [4.9-12.6]	2.16 [0.75-4.48]	2.04 [0.82-2.92]	<0.001	0.28

CTEPH, chronic thromboembolic pulmonary hypertension; ET-1, endothelin 1; hsCRP, high sensitivity C-reactive protein; IL-6, interleukin 6; IL-8, interleukin 8; IL-10, interleukin 10; MCP-1, monocyte chemoattractant protein 1

[a] – before vs after single BPA; [b] – before and after completion of the treatment

Table S2. Comparison of serum concentration of selected biomarkers between treatment naive patients with CTEPH, patients with CTEPH on treatment and control group.

	CTEPH (n=9) treatment naive	CTEPH (n=11) on treatment	Controls (n=10)	p*	p#
hsCRP	1.46 [0.71-3.7]	2.4 [0.79-7.33]	2.15 [0.88-2.77]	0.44	0.87
IL-6	4.79 [3.55-5.51]	3.76 [2.42-6.55]	1.81 [0.88-4.48]	0.57	0.03
IL-8	15.23 [13.55-19.1]	22.19 [14.43-42.88]	13.45 [12.46-16.02]	0.16	0.46
IL-10	0.51 [0.4-0.84]	0.53 [0.44-0.58]	0.51 [0.48-0.69]	0.75	0.79
MCP-1	445 [339-487]	417 [337-525]	416 [273-505]	0.79	0.83
ET-1	2.77 [1.7-3.49]	2.6 [2.31-3.81]	1.47 [1.4-1.82]	0.38	0.03

CTEPH, chronic thromboembolic pulmonary hypertension; ET-1, endothelin 1; hsCRP, high sensitivity C-reactive protein; IL-6, interleukin 6; IL-8, interleukin 8; IL-10, interleukin 10; MCP-1, monocyte chemoattractant protein 1

* - treatment naive vs. on treatment; #- treatment naïve vs. controls
